# Bioavailability of Glucoraphanin and Sulforaphane from High‐Glucoraphanin Broccoli

**DOI:** 10.1002/mnfr.201700911

**Published:** 2018-03-08

**Authors:** Tharsini Sivapalan, Antonietta Melchini, Shikha Saha, Paul W. Needs, Maria H. Traka, Henri Tapp, Jack R. Dainty, Richard F. Mithen

**Affiliations:** ^1^ Food and Health Programme Quadram Institute Bioscience Norwich United Kingdom; ^2^ Analytical Support Unit Quadram Institute Bioscience Norwich United Kingdom

**Keywords:** bioavailability, broccoli, glucoraphanin, pharmacokinetics, sulforaphane

## Abstract

**Scope:**

Broccoli accumulates 4‐methylsulphinylbutyl glucosinolate (glucoraphanin) which is hydrolyzed to the isothiocyanate sulforaphane. Through the introgression of novel alleles of the Myb28 transcription factor from *Brassica villosa*, broccoli genotypes have been developed that have enhanced levels of glucoraphanin. This study seeks to quantify the exposure of human tissues to glucoraphanin and sulforaphane following consumption of broccoli with contrasting Myb28 genotypes.

**Methods and results:**

Ten participants are recruited into a three‐phase, double‐blinded, randomized crossover trial (NCT02300324), with each phase comprising consumption of 300 g of a soup made from broccoli of one of three Myb28 genotypes (Myb28^B/B^, Myb28^B/V^, Myb28^V/V^). Plant myrosinases are intentionally denatured during soup manufacture. Threefold and fivefold higher levels of sulforaphane occur in the circulation following consumption of Myb28^V/B^ and Myb28^V/V^ broccoli soups, respectively. The percentage of sulforaphane excreted in 24 h relative to the amount of glucoraphanin consumed varies among volunteers from 2 to 15%, but does not depend on the broccoli genotype.

**Conclusion:**

This is the first study to report the bioavailability of glucoraphanin and sulforaphane from soups made with novel broccoli varieties. The presence of one or two Myb28^V^ alleles results in enhanced delivery of sulforaphane to the systemic circulation.

## 1 Introduction

Observational studies suggest that diets rich in cruciferous vegetables such as broccoli are associated with a reduction in the risk of cardiovascular disease and cancer.^1^ These health benefits have been attributed to degradation products of glucosinolates—specialized sulfur‐containing glycosides that accumulate within these vegetables.^2^, ^3^, ^4^ These degradation products are generated either due to the action of plant thioglucosidases (‘myrosinases’) that also occur within these vegetables, but which remain spatially separated from glucosinolates in the absence of tissue disruption, or, if these enzymes have been denatured due to cooking, by microbial thioglucosidases within the human gastrointestinal tract.^5^ Glucosinolates possess an aglycone side chain derived from an amino acid; glucosinolates derived from methionine generate isothiocyanates (ITCs) upon hydrolysis, while those derived from tryptophan generate a small number of indole compounds.^6^ Both ITCs and indoles have been associated with health‐ promoting effects.^3^ The most abundant glucosinolate in broccoli is the methionine‐derived 4‐methylsulfinylbutyl glucosinolate (glucoraphanin) that generates the isothiocyanate sulforaphane upon hydrolysis. This nonvolatile ITC has been extensively studied in model systems, and been associated with many biological processes that may underpin the health benefits attributed to cruciferous vegetables.^4^ Foremost among these is the potent induction of the nuclear factor (erythroid‐derived 2)‐like 2 (nrf2) transcription factor that regulates the expression of phase 2 detoxification and ‘antioxidant’ genes. Following absorption, sulforaphane is metabolized through the mercapturic acid pathway and excreted as predominantly an N‐acetyl cysteine conjugate in urine. Glucoraphanin and sulforaphane may be reduced to their methylthiobutyl analogue, commonly known as glucoerucin and erucin respectively, either through enzymic activity by the gut microbiota^5^ or nonenzymically through changes in the redox environment.^7^

The level of sulforaphane that human tissue will be exposed to following consumption of broccoli is dependent upon several factors. The most important of these are the absolute amount of the precursor glucoraphanin in the broccoli itself; the hydrolysis of glucoraphanin by plant myrosinases or the gut microbiota; and the extent of absorption from the upper and lower gastrointestinal tract. Studies with the model plant *Arabidopsis* and with *Brassica* have identified the Myb28 transcription factor as having an important regulatory role in the expression of glucoraphanin, through modulating the expression of genes associated with sulfate assimilation and glucosinolate biosynthesis.^8^, ^9^, ^10^ Moreover, it has been shown that the Myb28 allele from the related *Brassica* species *Brassica villosa* (Myb28^v^) when introgressed into a broccoli genetic background to replace the normal broccoli allele (Myb28^B^) results in an enhanced expression of Myb28 mRNA and greater accumulation of methionine‐derived glucosinolates.^8^

In this paper, we seek to explore the consequences of the introgression of one or two Myb28^v^ alleles into broccoli on the pharmacokinetics of glucoraphanin and sulforaphane following consumption through a three‐phase, double blinded, randomized crossover trial. To simulate likely domestic cooking scenarios, we denature all endogenous plant myrosinases through blanching the broccoli prior to developing a broccoli soup through a standard commercial manufacturing process.

## 2 Experimental Section

### 2.1 Materials

BD Vacutainer EDTA (purple capped) blood collection tubes were purchased from Becton Dickinson and Company. DNeasy blood & tissue kit and RNase (100 mg mL^–1^) were acquired from Qiagen Ltd. TaqMan Drug Metabolism SNP Genotyping Assays and TaqMan Universal Master Mix II without uracil‐N‐glycosylase (UNG) were purchased from Thermo Fischer Scientific. Sulforaphane (CAS 4478‐93‐7) (purity > 98%) was purchased from LKT Laboratories. The internal standard N‐butylthiocarbamoyl cysteine (B‐ITC) and sulforaphane conjugates including sulforaphane‐glutathione, sulforaphane‐cysteine‐glycine, sulforaphane‐cysteine, sulforaphane‐N‐acetylcysteine (‐NAC), and erucin‐NAC were synthesized as published in.^11^ The internal standard sinigrin for glucoraphanin was purchased from Sigma–Aldrich. Blank plasma from healthy participants with the same diet restriction described in ‘Study design’ was ordered from Sera Laboratories International Ltd to make standard curves. DEAE Sephadex A25 and SP Sephadex C25 were obtained from Amersham Biosciences. Glucoraphanin (CAS 21414‐41‐5) (purity ≥ 95%) and glucoerucin (CAS 21973‐56‐8) (purity ≥ 97%) were purchased from Cayman Chemical and from Carl Roth, respectively. All other chemicals were purchased from Sigma–Aldrich.

### 2.2 Study Design

Men and woman aged 18–65 years with a BMI between 19.5 and 35 kg m^–2^ were enrolled into a randomized, double‐blinded, three‐phase crossover trial carried out at the Human Nutrition Unit (HNU) at the Quadram Institute Bioscience (QIB). Participants were recruited on the basis of fasted (≥8 h) screening blood/urine samples and a completed health questionnaire. With the use of an electronic randomization generator (http://www.randomization.com), a randomization sequence was generated prior to the start of the intervention allocation. Each phase involved a 48‐h preintervention diet restriction, a study day involving a 9 h stay at the HNU and a sample collection the following morning (24 h post soup consumption) followed by a washout period of 2 weeks. Participants (*n* = 10) were required to follow a glucosinolate‐free diet as well as avoiding alcohol, spicy food and garlic for 48 h prior to each study day until the collection of the 24 h sample for each phase. This dietary restriction was required to ensure that glucosinolates from other food sources did not have an impact on the study results. During each study day, 11 blood samples (10 mL) were collected after the consumption of the soups at the following timepoints: 0, 30, 45, 60, 90, 120, 180, 240, 360, 480 min, and 24 h. Six urine samples were collected at the following timepoints: 0, 0–2, 2–4, 4–6, 6–8, and 8–24 h. Ethical approval was obtained from the Human Research Governance Committee at QIB (IFR06/2014) and the NRES East of England Norfolk Ethics Committee (14/EE/1121). The study was registered on Clinicaltrials.gov (NCT02300324). Written informed consent of all participants was obtained. The study lasted from September 2014–August 2015.

### 2.3 Dietary Intervention

Participants were randomly allocated to one of the three types of broccoli soups at each phase: (i) 300g Myb28^B/B^ broccoli (standard broccoli) and stilton soup, (ii) 300g Myb28^B/V^ broccoli (Beneforte) and stilton soup, and (iii) 300g Myb28^V/V^ broccoli and stilton soup. Glucoraphanin levels in soups were quantified using a validated HPLC–DAD method as described in Saha et al.^12^ involving the conversion of glucosinolates to desulfoglucosinolates. The soups were manufactured by Bakkavor (Lincolnshire, UK) and frozen immediately. In order to avoid variability in the cooking process, the study team implemented a standard operating procedure that involved heating the study diet in the microwave at the HNU for ≈6 min on the morning of the study phases. All three types of soups appeared the same and had the same flavor enabling the blinding for both study scientists and participants.

### 2.4 Biological Sample Collection and Processing

Blood samples (10 mL) were collected from the intravenous catheter into EDTA (BD Vacutainer). Aliquots of whole blood were immediately placed in dry ice for storage at –80 °C for genotyping. Whole blood was also centrifuged at 1000 × *g* for 15 min at room temperature and plasma was aliquoted and stored at –80 °C for analysis of metabolites. Plasma samples were extracted on ice by adding 20 μL of precooled 50% trichloroacetic acid (glucosinolate analysis) or trifluoroacetic acid (sulforaphane analysis), and 10 μL of internal standard (B‐ITC or sinigrin) to 100 μL of plasma sample. The samples were vortexed for 30 s followed by centrifugation at 17 000 × *g* at 4 °C for 10 min. The supernatant was transferred into Thermo Scientific insert vials and analyzed on the UPLC–MS/MS and LC–MS/MS for glucoraphanin and sulforaphane, respectively.

Filtered urine aliquots (100 μL) were extracted by the addition of 890 μL of either ammonium acetate buffer and 10 μL of internal standard (B‐ITC) for sulforaphane, or 0.5% formic acid and 10 μL of internal standard (sinigrin) for glucoraphanin. Samples were vortexed for 30 s then centrifuged for 10 min at 17 000 × *g* at 4 °C. The supernatant was transferred into Agilent vials and analyzed on the UPLC–MS/MS and Agilent 6490 Triple Quad LC–MS/MS system for glucoraphanin and sulforaphane, respectively.

### 2.5 UPLC–MS/MS Analysis of Glucoraphanin in Plasma and Urine Samples

We developed a novel and highly sensitive method to accurately detect levels of glucoraphanin and its reduced analogue glucoerucin from plasma and urine samples.

Glucoraphanin and glucoerucin analysis was performed on an Agilent 6490 tandem mass spectrometer equipped with an Agilent 1260 HPLC system (Agilent Technologies, Santa Clara, CA, USA). The chromatographic separation of glucoraphanin, glucoerucin and sinigrin was conducted on a Kinetex 1.7 μm XB‐C18 100 Å 100 × 2.1 mm UPLC column. The mobile phase was composed of MilliQ water containing 0.2% formic acid (mobile A) and acetonitrile containing 0.2% formic acid (mobile B). The gradient started at 5% mobile phase B increasing over 7 min to 80% mobile phase B and finally reequilibrating to 5% mobile phase B for 2 mi. The total run time was 10 min, and the flow rate was 300 μL min^–1^. The LC eluent flow was sprayed into the mass spectrometer interface without splitting. The MS/MS system was equipped with an ESI source operated in negative‐ion detection mode. Nitrogen gas was used for nebulation, desolvation, and collision. The analytes were monitored in multiple‐reaction monitoring (MRM) mode. The MRM precursor, product ions, and collision energy were optimized by Agilent optimizer software. The transitions of precursor ions to product ions (*m/z)* and some optimized MS operating parameters of the analytes are described in Supporting Information Table S1. Other settings for the mass spectrometer were as follows: gas flow 16 L min^–1^ at 200 °C, nebulizer pressure 50 psi, sheath gas flow 11 L min^–1^ at 300 °C, and capillary voltage 3500 V. Quantification was performed using the ratio of the integrated peak area of glucoraphanin and glucoerucin to that of sinigrin and was calculated with MassHunter Workstation software (version B.06, Agilent Technologies).

Matrix match calibration curve was used to plot the ratios of the analyte peak area to the sinigrin peak area at nine different concentrations of authentic standard ranging from 0.0 to 2.0 μg mL^–1^ for both glucoraphanin and glucoerucin. The linearity of the response was assessed by means of least‐squares linear regression.

### 2.6 LC–MS/MS Analysis of Sulforaphane and its Conjugates in Plasma and Urine Samples

Sulforaphane and conjugates were analyzed in plasma and urine samples as described before^13^ with slight modifications. Metabolites were separated on the HPLC Phenomenex Luna 3u C18 (2) 100A 100 × 2.1 mm column with a flow of 0.25 mL min^–1^. All MRM transitions (precursor and products ions) and collision energy were optimized by Agilent optimizer software. The source parameters included a gas temperature at 200 ˚C with a gas flow of 12 L min^–1^, a sheath gas temperature at 400 ˚C with a sheath gas flow of 12 L min^–1^, a nebuliser pressure of 60 psi, and capillary voltage at 4000 V. In addition, erucin‐NAC was also measured by using the same method.

### 2.7 Genotyping

Genomic DNA was extracted from 100 μL whole blood samples using the Qiagen DNeasy blood & tissue kit from Qiagen as per manufacturer's instructions. The TaqMan Drug Metabolism SNP Genotyping Assay for GSTM1 (assay ID: C4420299720) was used to determine glutathione S‐transferase mu 1 (GSTM1) gene deletion and allelic variance, using the AB StepOnePlus instrument. Data were quantified relative to a two‐copy gene control, a region in IVS10 of the breast cancer 1, early onset (BRCA1, NM_007294) gene.^14^

### 2.8 Statistical Analysis

The study was powered to detect a significant (p<0.05) difference in mean total urinary thiol excretion of 18 μmol between consumption of standard broccoli (Myb28^B/B^) and stilton soup and Beneforte® broccoli (Myb28^V/B^) and stilton soup, with a power of 80%. Pharmacokinetics parameters of the plasma data (AUC), T_max_ (time of maximum concentration), C_max_ (maximum concentration) were calculated on Prism (GraphPad Software, La Jolla California USA, http://www.graphpad.com). Urine data were analyzed to give total excreted (Total Excrete) and the percentage excreted based on the knowledge of the soup compositions (Total Excrete %). Response variables were modeled using sequential analysis of variance. Standard model diagnostics were applied for example transformations of the response or removal of potential outliers. Significance levels for multiple comparisons are based on Tukey's honest significant difference. The statistician was blinded during the analysis of the data.

## 3 Results

### 3.1 Subject Recruitment

**Figure 1:**
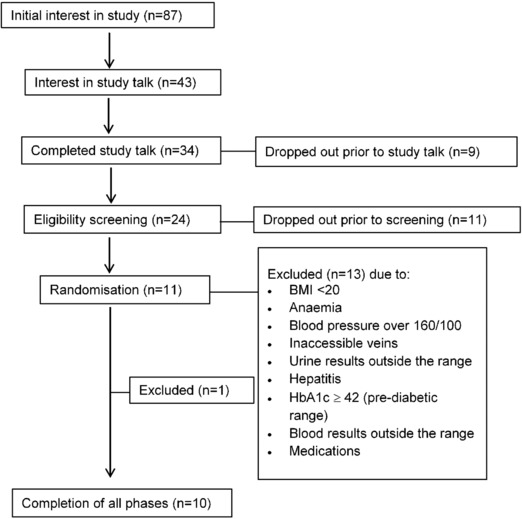
Flow diagram for participant recruitment.

Ten participants (seven females and three males) were recruited into the study (**Figure** 1) and completed all three phases without any adverse effects. The participants had the following baseline characteristics (age 42.9 ± 17.4 years, height 170.9 ± 10.6 cm, weight 73.6 ±13.6 kg, BMI 25.2 ± 3.2 (kg m^–^²). Out of ten participants, six were *GSTM1*‐positive (either homozygous or heterozygous for a functional GSTM1 allele), and four were *GSTM1*‐null. The 40% occurrence of *GSTM1*‐null genotype in this small study sample is within the expected range of 39–62% for Caucasians.^15^

### 3.2 Glucosinolate Levels in the Intervention Soups

**Figure 2:**
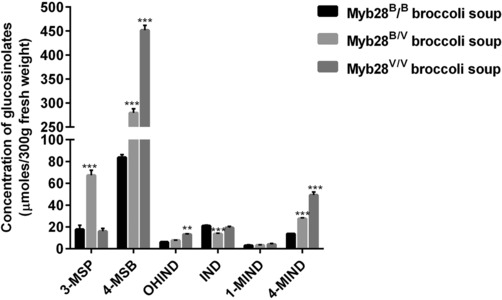
Concentration of glucosinolates in the soups with different broccoli genotypes. Glucosinolates measured includes glucoraphanin (4‐MSB, 4‐methylsulphinylbutyl), 3‐methylsulphinylpropyl (3‐MSP), indolylmethyl (IND), 1‐hydroxy‐indolylmethyl (OHIND), 1‐methoxy‐indolylmethyl (1‐MIND), and 4‐methoxy‐indolylmethyl (4‐MIND). Data are represented as mean ± SD (*n* = 10, Myb28^B/B^, Myb28^B/V^ soups; *n* = 4, Myb28^V/V^ soups). Statistical analysis of the data was undertaken by two‐way analysis of variance with Dunnett's multiple comparisons test (**p ˂ 0.01 and ***p˂0.0001 vs Myb28^B/B^ broccoli soup).

The glucosinolate analyses of the three types of broccoli soups were consistent with their Myb28 genotype. Among the glucosinolates derived from broccoli, the predominant glucosinolate was glucoraphanin (**Figure** 2). Myb28^V/V^ and Myb28^B/V^ broccoli soups contained 452 ± 10.6 μmoles glucoraphanin per 300 mL portion and 280 ± 8.8 μmoles glucoraphanin per 300 mL portion respectively, approximately five‐ and threefold greater glucoraphanin levels compared to Myb28^B/B^ broccoli soup that contained 84 ± 2.8 μmoles glucoraphanin per 300 mL. Glucoerucin, sulforaphane, and erucin were not present in the soups.

### 3.3 Pharmacokinetic Profile of Glucoraphanin and its Reduced Analogue

**Figure 3:**
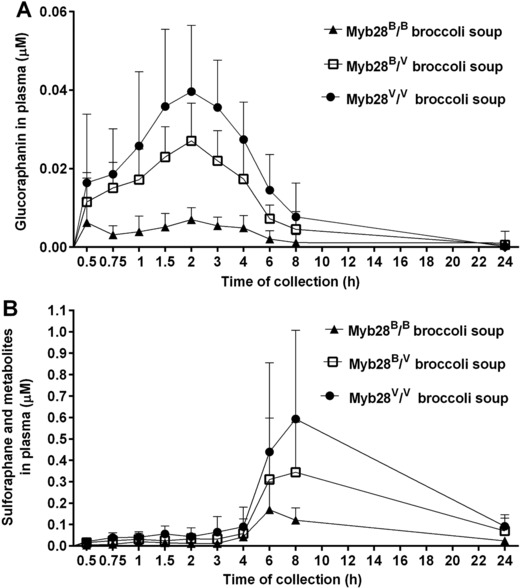
Plasma concentrations (μm) of intact glucoraphanin (A) and sulforaphane (B) following consumption of Myb28^B/B^ (84 μmoles glucoraphanin per 300 g soup), Myb28^B/V^ (280 μmoles glucoraphanin per 300 g soup), and Myb28^V/V^ (452 μmoles glucoraphanin per 300 g soup) broccoli soups. The samples were analyzed by UPLC–MS/MS and LC–MS/MS for glucoraphanin and sulforaphane, respectively. Samples were analyzed for sulforaphane metabolites including sulforaphane‐glutathione, sulforaphane‐cysteine, sulforaphane–cysteine‐glycine, and sulforaphane‐NAC. Data (*n* = 10) are represented as mean ± SD.

**Figure 4:**
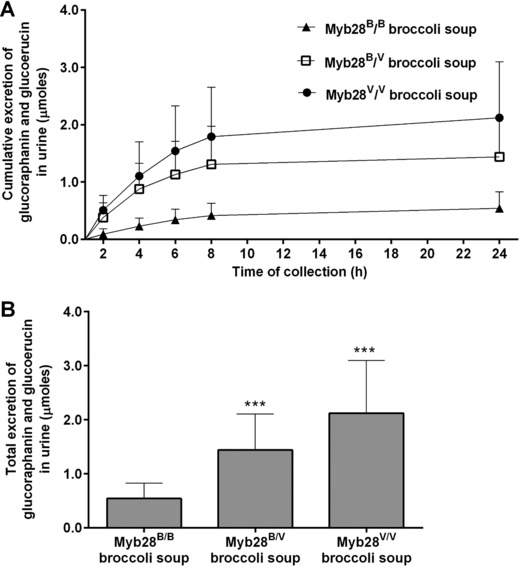
Urinary excretion of intact glucoraphanin and glucoerucin (μmoles) in 24 h following consumption of Myb28^B/B^ (84 μmoles glucoraphanin per 300 g soup), Myb28^B/V^ (280 μmoles glucoraphanin per 300 g soup), and Myb28^V/V^ (452 μmoles glucoraphanin per 300 g soup) broccoli soups. Cumulative excretion of glucoraphanin/glucoerucin (A), and the total urinary excretion of glucoraphanin/glucoerucin (B) measured in urine by UPLC–MS/MS. Data (*n* = 10) are represented as mean ± SD. Total urinary excretion data underwent square root transformation followed by analysis by one‐way analysis of variance with Tukey's honest significance test (***p ˂ 0.0001 vs My28^B/B^ broccoli soup).

Plasma and urine samples collected from participants were analyzed by UPLC–MS/MS to assess the bioavailability of unmetabolized glucoraphanin and its reduced analogue glucoerucin. Glucoraphanin was detected in both plasma and urine samples, and both glucoraphanin and glucoerucin were found in urine. Glucoraphanin was detectable in plasma within 30 min, peaked at 2 h and then decreased to undetectable levels by 24 h (**Figure** 3A, **Table** 1). The AUC and C_max_ for glucoraphanin from broccoli with either one or two Myb28^V^ alleles was significantly greater compared to broccoli without Myb28^V^ alleles (*p* < 0.0001, Table 1). Glucoraphanin and glucoerucin were detected in urine within 2 h after consumption from all three types of broccoli soups. The cumulative amount of glucoraphanin and glucoerucin increased until 8 h after consumption (**Figure** 4A). A greater amount of glucoraphanin was excreted in the urine following consumption of the soups with either one or two Myb28^V^ alleles (Figure 4B).

**Table 1:** Summary table of the levels of unmetabolized glucoraphanin and its reduced analogue in plasma and urine after consumption of soups with different broccoli genotype

	Myb28^B/B^ broccoli soup	Myb28^B/V^ broccoli soup	Myb28^V/V^ broccoli soup	*p*‐Value for broccoli genotypes			
	Mean ± SD	Mean ± SD	Mean ± SD	Analysis of variance	Myb28^B/B^ versus Myb28^B/V^	Myb28^B/B^ versus Myb28^V/V^	Myb28^B/V^ versus Myb28^V/V^
Glucoraphanin in plasma							
AUC (μmol h L^–1^)	0.05 ± 0.05	0.15 ± 0.08	0.24 ± 0.11	<0.0001	<0.0001	<0.0001	<0.05
C_max_ (μmol L^–1^)	0.01 ± 0.01	0.03 ± 0.01	0.04 ± 0.02	<0.0001	<0.0001	<0.0001	<0.01
T_max_ (h)	2.20 ± 1.16	2.23 ± 0.90	2.25 ± 0.95	0.8006			
Concentration at 24 h (μmol L^–1^)	˂0.1	˂0.1	˂0.1	0.8389			
Glucoraphanin and glucoerucin in urine							
Total excreted in 24 h (μmoles)	0.54 ± 0.29	1.44 ± 0.66	2.12 ± 0.98	<0.0001	<0.0001	<0.0001	<0.05
Percentage excreted 24 h after ingestion %	0.64 ± 0.34	0.51 ± 0.24	0.47 ± 0.22	0.6053			

Analysis modeled using sequential analysis of variance. The variables including AUC, total excreted, and percentage excreted underwent a square root transformation, Cmax and Tmax underwent a log transformation. Concentration at 24 h underwent a transformation of (y + 9.5463e‐05)^–1^. Significance level for mutliple comaprisons was analyzed using Tukey's honest significance test.

### 3.4 Pharmacokinetics of Sulforaphane and its Conjugates

**Figure 5:**
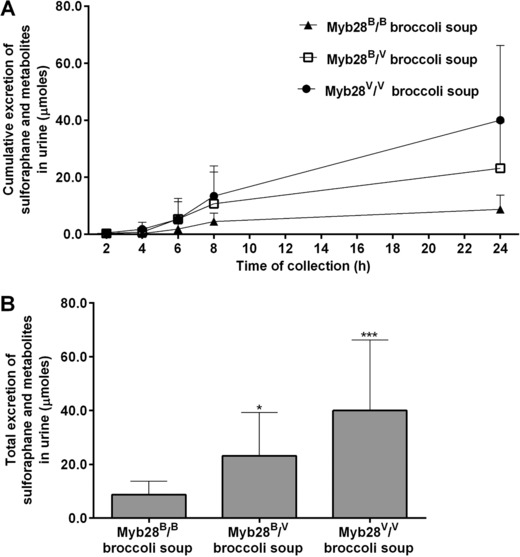
Urinary excretion of sulforaphane and metabolites (μmoles) in 24 h following consumption of Myb28^B/B^ (84 μmoles glucoraphanin per 300 g soup), Myb28^B/V^ (280 μmoles glucoraphanin per 300 g soup), and Myb28^V/V^ (452 μmoles glucoraphanin per 300 g soup) broccoli soups. Cumulative excretion (A) and total urinary excretion (B) of sulforaphane and metabolites in urine samples analyzed by LC–MS/MS. Samples were analyzed for sulforaphane metabolites including erucin‐NAC, sulforaphane‐cysteine, sulforaphane–cysteine‐glycine, and sulforaphane‐NAC. Data (*n* = 10) are represented as mean ± SD. Total urinary excretion data underwent log root transformation followed by analysis by one‐way analysis of variance with Tukey's honest significance test (*p < 0.05 and ***p ˂ 0.001 vs My28B/B broccoli soup).

Within 30 min of consumption of all three types of soups, sulforaphane, its glutathione conjugate, and derivatives (‘sulforaphane metabolites’) were detected in plasma samples (**Table** 2, Figure 3B). T_max_ occurred between 6 and 9 h following consumption. AUC and C_max_ were significantly greater for broccoli possessing one or two Myb28^V^ alleles compared to standard broccoli (**Table** 3). Approximately 35% of sulforaphane occurred unconjugated in the plasma (Table 2). Within 1 h of consumption, sulforaphane and metabolites were detected in the urine (**Figure** 5). The cumulative amount of sulforaphane and metabolites excreted in the urine was significantly higher following consumption of the soups with Myb28^V/V^ and Myb28^B/V^ than with Myb28^B/B^ (Figure 5A, Table 3).

**Table 2:** Summary table of the percentage of individual sulforaphane metabolites in plasma and urine after consumption of soups with different broccoli genotypes

	Myb28^B/B^ broccoli soup	Myb28^B/V^ broccoli soup	Myb28^V/V^ broccoli soup
	Mean ± SD (%)	Mean ± SD (%)	Mean ± SD (%)
Metabolites in plasma over 24 h			
Sulforaphane	32.20 ± 17.29	34.59 ± 14.67	36.58 ± 14.35
Sulforaphane‐glutathione	34.57 ± 21.74	33.01 ± 23.80	30.45 ± 21.21
Sulforaphane‐cysteine	8.93 ± 9.93	5.54 ± 3.53	8.77 ± 8.80
Sulforaphane–cysteine‐glycine	21.60 ± 11.81	22.22 ± 10.27	20.23 ± 7.38
Sulforaphane‐NAC	2.70 ± 1.79	4.63 ± 4.29	3.97 ± 2.84
Metabolites in 24‐h urine excretion			
Sulforaphane	8.53 ± 5.57	5.66 ± 3.04	5.41 ± 2.20
Sulforaphane‐NAC	57.78 ± 11.73	49.46 ± 14.08	51.26 ± 11.07
Sulforaphane‐cysteine	18.29 ± 6.18	19.15 ± 9.20	21.08 ± 5.61
Erucin‐NAC	20.93 ± 9.04	25.30 ± 16.73	21.15 ± 13.51
Sulforaphane‐cysteine‐glycine	0.48 ± 0.90	0.44 ± 0.72	1.11 ± 2.74

Data (*n* = 10) is represented as mean ± SD. The percentage was calculated as the concentration of each individual metabolite (μm) of the total concentration of sulforaphane and metabolites (μm).

**Table 3:** Summary table of the levels of sulforaphane and metabolites in plasma and urine after consumption of soups with different broccoli genotypes

	Myb28^B/B^ broccoli soup	Myb28^B/V^ broccoli soup	Myb28^V/V^ broccoli soup	*p*‐Value for broccoli soup			
	Mean ± SD	Mean ± SD	Mean ± SD	Analysis of variance	Myb28^B/B^ versus Myb28^B/V^	Myb28^B/B^ versus Myb28^V/V^	Myb28^B/V^ versus Myb28^V/V^
Sulforaphane and metabolites in plasma							
AUC (μmol h L^–1^)	1.99 ± 1.31	4.92 ± 3.77	8.08± 6.60	<0.0001	˂0.01	<0.0001	0.0905
C_max_ (μmol L^–1^)	0.17 ± 0.12	0.37 ± 0.26	0.61 ± 0.40	≤0.0001	˂0.01	≤0.0001	0.0683
T_max_ (h)	6.10 ± 2.23	7.40 ± 0.97	9.20 ± 5.27	˂0.05	0.5258	˂0.05	0.2301
Concentration at 24 h (μmol L^–1^)	0.02 ± 0.01	0.07 ± 0.06	0.09 ± 0.05	<0.0001	˂0.01	<0.0001	0.1797
Sulforaphane and metabolites in urine							
Total excreted in 24 h (μmoles)	8.74 ± 4.95	23.14 ± 16.17	39.98 ± 26.22	<0.001	˂0.05	<0.001	˂0.01
Percentage excreted 24 h after ingestion %	10.40 ± 5.90	8.26 ± 5.78	8.85 ± 5.80	0.5727			

Analysis modeled using sequential analysis of variance. The variables underwent log transformation except for plasma concentration at 24 h, which underwent a square root transformation. Significance level for multiple comparisons was analyzed using Tukey's honest significance test. ‘sulforaphane metabolites’ refers to sulforaphane, sulforaphane‐glutathione, sulforaphane‐cysteine, sulforaphane–cysteine‐glycine, and sulforaphane‐NAC in plasma and sulforaphane, sulforaphane‐NAC, sulforaphane‐cysteine, erucin‐NAC, and sulforaphane–cysteine‐glycine in urine.

To explore whether the *GSTM1* genotype influenced the metabolism of sulforaphane and metabolites, pharmacokinetic parameters were stratified according to *GSTM1* genotypes. There was no significance on the plasma pharmacokinetics (AUC, C_max_, and T_max_) and the urinary excretion between *GSTM1*‐null and *GSTM1*‐positive participants (*p* > 0.05, data not shown).

### 3.5 Inter‐Individual Variation in the Percentage Urinary Excretion of Glucoraphanin and Sulforaphane

**Figure 6:**
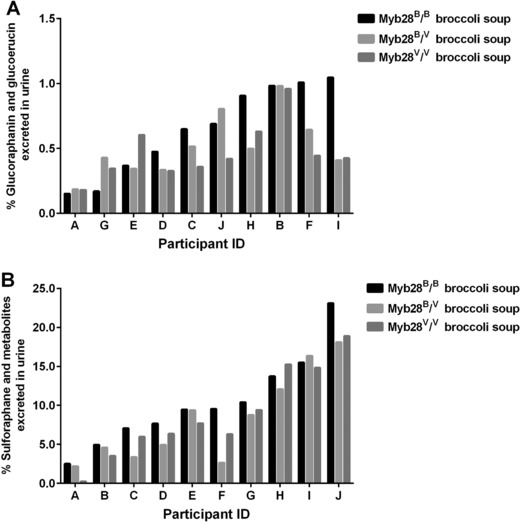
Percentage of glucoraphanin (A) and sulforaphane (B) excreted in the urine following consumption of the ingested dose of glucoraphanin in the broccoli soups, Myb28^B/B^ (84 μmoles glucoraphanin per 300 g soup), Myb28^B/V^ (280 μmoles glucoraphanin per 300 g soup), and Myb28^V/V^ (452 μmoles glucoraphanin per 300 g soup). Urine samples collected from participants (A–J) were analyzed for glucoraphanin, glucoerucin, sulforaphane, and its metabolites including sulforaphane, erucin‐NAC, sulforaphane‐cysteine, sulforaphane–cysteine‐glycine, and sulforaphane‐NAC.

The percentage urinary excretion, represented as the amount of sulforaphane and metabolites excreted in the urine relative to the glucoraphanin consumed, exhibited considerable variation between individuals but was relatively constant for any one individual regardless of which broccoli genotype was consumed, varying from ≈15% for participant J to less than 3% from participant A. Likewise, there was variation among individuals in their excretion of glucoraphanin as a percentage of intake (**Figure** 6). There was not a significant correlation between the % excreted as sulforaphane and as glucoraphanin, suggesting that these were determined by different metabolic processes (Supporting Information Figure S1).

## 4 Discussion

Observational studies suggest that people who consume more than four or five portions of cruciferous vegetables per week have reduced risk of cancer at a number of sites and other chronic disease.^1^, ^16^, ^17^, ^18^, ^19^, ^20^ A multitude of studies in cell and animal models have implicated the ITC sulforaphane in these health‐promoting effects,^4^ despite very little evidence in humans. To facilitate human studies that could attempt to dissect the effect of sulforaphane from other nutritional factors within broccoli, novel broccoli Myb28 genotypes have been developed with enhanced levels of the sulforaphane precursor, glucoraphanin. The aim of this study was to quantify the pharmacokinetics of sulforaphane derived from these novel broccoli genotypes in the absence of any plant‐derived myrosinase activity, and, in so doing, evaluate whether the enhanced glucoraphanin content in the plant tissue and processed food results in enhanced levels of sulforaphane in plasma. In addition, we sought to investigate whether any of the unmetabolized glucoraphanin is absorbed, facilitated through the development of a new UPLC–MS/MS method as described above.

For both C_max_ and AUC, the level of sulforaphane and its metabolites in plasma was approximately fivefold higher following consumption of soup from the homozygous Myb28^V/V^ broccoli genotype, and threefold higher for the heterozygous Myb28^V/B^ broccoli genotype, compared to the standard Myb28^B/B^ broccoli (Figure 3B, Table 1). These values reflect the differences in the glucoraphanin content of the broccoli. Intact glucoraphanin was also detected in the plasma and urine and, as with sulforaphane, the relative amounts in plasma and urine reflected the levels that were present in the broccoli (Figure 3A and 3B). The T_max_ for glucoraphanin of ≈2 h is indicative of absorption from the upper GI tract, in contrast to the much later T_max_ for sulforaphane indicative of absorption from the lower GI tract following microbial metabolism of glucoraphanin (Figure 3A and 3B). The excretion of sulforaphane as a percentage of glucoraphanin consumed varied between 2 and 15%. The consistent nature of this value for each volunteer regardless of which broccoli genotype they consumed is notable (Figure 6). The variation among volunteers is likely due to differences in their gut microbiota and their ability to metabolize glucoraphanin.

The results from the current study are consistent with that reported by Gasper at al.^21^ In this previous study, glucoraphanin was converted to sulforaphane prior to consumption, unlike the current study, and it was shown that consuming a threefold greater amount of sulforaphane led to a threefold greater level in plasma. In this case, as may be expected, T_max_ occurred after about 2 h due to the lack of a need for microbial metabolism of glucoraphanin, and a larger percentage of the consumed sulforaphane (40–90%) was accounted for in the urine after 24 h. This previous study also indicated that *GSTM1* genotype was a factor in determining the extent of sulforaphane excretion. This was not observed in the current study. This may be because the inter‐individual variation in glucoraphanin conversion by the gut microbiota to sulforaphane obscures any potential effect of genotype, and a much larger study would be required to detect any effect of *GSTM1*. The expected C_max_ of sulforaphane in the current study from the standard broccoli was higher than that which might have been expected based upon studies of Saha et al.^12^ and Vermeulen et al.^22^ This may be due to the use of a different food matrix as the soups used in the current study were rich in fat and other nutrients that may facilitate greater release of glucoraphanin from plant tissue within the gastrointestinal tract

Previous studies have also reported the presence of unconjugated sulforaphane and its thiol conjugates in plasma and urine, as reported in the present study, although there are differences in its relative abundance compared to its thiol conjugates.^12^, ^23^ Consistent with other findings, the most abundant metabolite excreted in urine was sulforaphane‐NAC.^12^, ^21^, ^23^ Erucin‐NAC was the second major metabolite excreted with consumption of all three types of soups. Glucoerucin was not detected in the broccoli soups. The erucin‐NAC may be either derived from conversion of glucoraphanin to glucoerucin in the gastrointestinal tract and the subsequent generation of erucin ITC by the gut microbiota, or the reduction of sulforaphane post absorption.^5^, ^7^, ^12^

It is assumed that the glucoraphanin that was not accounted for by the urinary excretion of sulforaphane metabolites or glucoraphanin (and their reduced analogues) would have been excreted with faeces but further study is necessary.

In conclusion, this study has provided evidence that enhancing glucoraphanin content in broccoli through the simple introgression of *B. villosa* alleles of a single Myb28 gene results in enhanced exposure of human tissues to sulforaphane in a manner that may be expected to provide health benefits. This is maintained when glucoraphanin is delivered in a complex processed food, i.e. soups, and will allow for the future design of long‐term double‐blinded clinical studies to evaluate health benefits, with minimal changes to a participant's dietary habits.

**Abbreviations**
B‐ITC: N‐butylthiocarbamoyl cysteine
HNU: Human Nutrition Unit
ITCs: isothiocyanates
NAC: N‐acetylcysteine

## Conflict of Interest

The authors declare no conflict of interest.

## Supporting information


**Figure S1**: Correlation between the percentage of glucoraphanin and sulforaphane excreted of the ingested dose of glucoraphanin following consumption of Myb28^B/B^ (84 μmoles glucoraphanin per 300 g soup) **(A)**, Myb28^B/V^ (280 μmoles glucoraphanin per 300 g soup) **(B)** and Myb28^V/V^ (452 μmoles glucoraphanin per 300 g soup) **(C)**. Urine samples collected from participants (A–J) were analyzed for glucoraphanin, glucoerucin, sulforaphane, and its metabolites including erucin‐NAC, sulforaphane‐cysteine, sulforaphane–cysteine‐glycine, and sulforaphane‐NAC.

Table S1: LC–MS/MS parameters for glucoraphanin, glucoerucin, and sinigrin ions
