# Supplementary figures and images for: Bioavailability of Glucoraphanin and Sulforaphane from High‐Glucoraphanin Broccoli
Source: Mol Nutr Food Res. 2018 Mar 8;62(18):1700911. doi: 10.1002/mnfr.201700911 (PMC6175108; doi:10.1002/mnfr.201700911)

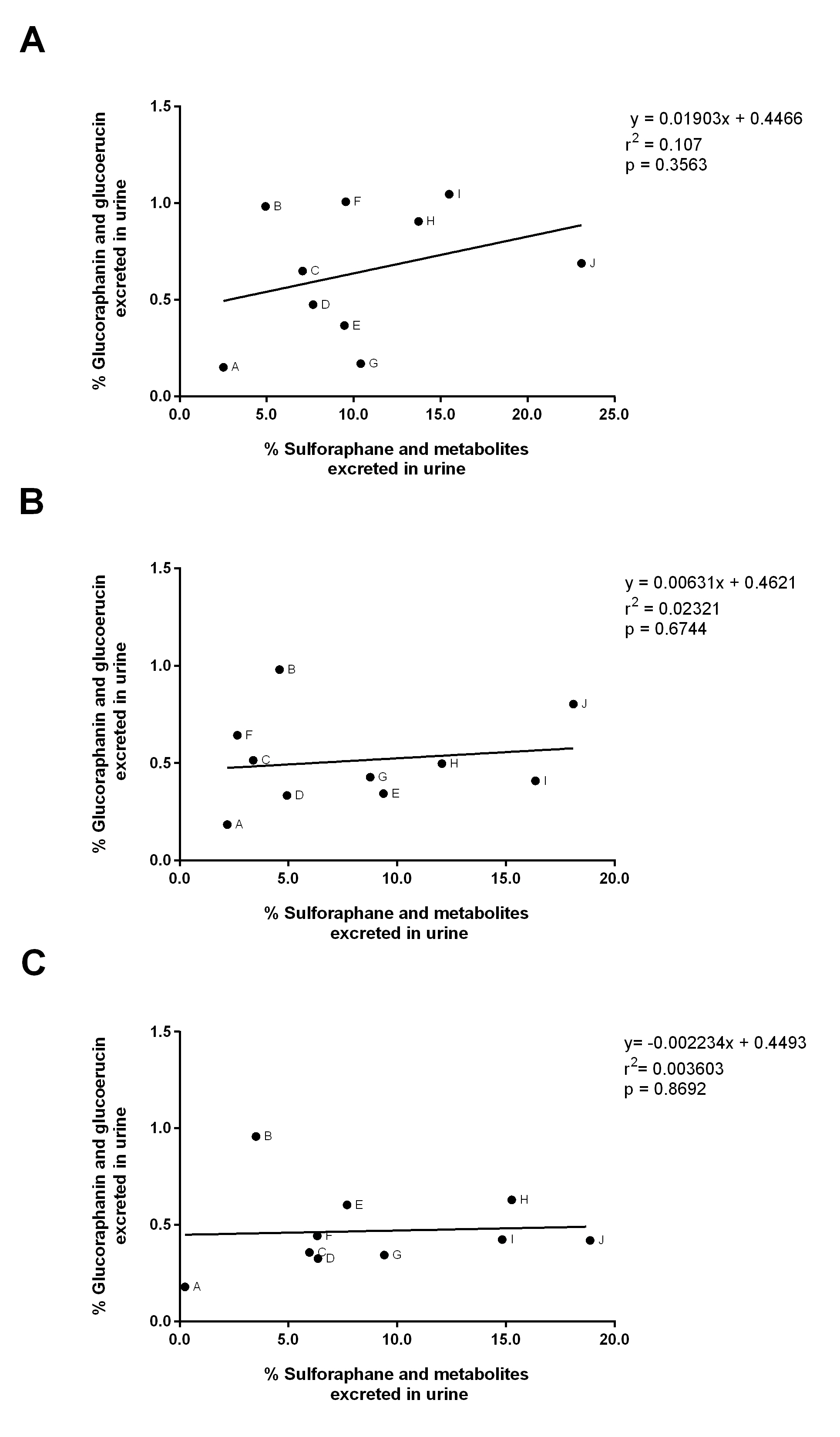

Supplement: Supplementary file 1 — Figure S1: Correlation between the percentage of glucoraphanin and sulforaphane excreted of the ingested dose of glucoraphanin following consumption of Myb28B/B (84 μmoles glucoraphanin per 300 g soup) (A), Myb28B/V (280 μmoles glucoraphanin per 300 g soup) (B) and Myb28V/V (452 μmoles glucoraphanin per 300 g soup) (C). Urine samples collected from participants (A–J) were analyzed for glucoraphanin, glucoerucin, sulforaphane, and its metabolites including erucin‐NAC, sulforaphane‐cysteine, sulforaphane–cysteine‐glycine, and sulforaphane‐NAC. [file MNFR-62-na-s001.jpg]
