# Supplementary material for: Bioavailability of Glucoraphanin and Sulforaphane from High‐Glucoraphanin Broccoli
Source: Mol Nutr Food Res. 2018 Mar 8;62(18):1700911. doi: 10.1002/mnfr.201700911 (PMC6175108; doi:10.1002/mnfr.201700911)
Supplement: Supplementary file 2 — Table S1: LC–MS/MS parameters for glucoraphanin, glucoerucin, and sinigrin ions [file MNFR-62-na-s002.docx]

Table S1: LC-MS/MS parameters for glucoraphanin, glucoerucin and sinigrin ions

| **Analyte** | **Retention time (mins)** | **Precrusor ion (m/z)** | **Product ion (m/z)** | **Collision energy** | **Cell accelerator energy** | **Dwell Time (ms)** |
| --- | --- | --- | --- | --- | --- | --- |
| Glucoraphanin | 2.0 | 436.42 | 372 | 18 | 4 | 80 |
| Glucoraphanin | 2.0 | 436.42 | 96.9 | 22 | 4 | 100 |
| Glucoerucin | 5.5 | 419.99 | 96.8 | 22 | 4 | 100 |
| Glucoerucin | 5.5 | 419.99 | 74.8 | 34 | 4 | 80 |
| Sinigrin | 2.3 | 357.99 | 96.9 | 22 | 4 | 100 |
